# Enhancing Peptide Hydrophilicity of SPPS-Derived Peptides Using Fmoc Noncanonical Amino Acids: A Review

**DOI:** 10.1021/acsbiomaterials.5c02185

**Published:** 2026-03-27

**Authors:** Wen Liu, Xing Wang, Rishi Pai, Jiahao Zhang, Christina Tran, Lei Li, Zhicheng Jin

**Affiliations:** Department of Chemistry, 1373 Georgia State University, Atlanta, Georgia 30303, United States

**Keywords:** solid-phase peptide synthesis, noncanonical amino acids, water solubility, side-chain engineering, Fmoc chemistry, biomaterials

## Abstract

Poor peptide solubility in water remains a major challenge in both peptide synthesis and downstream biomedical applications. Recent advances in 9-fluorenylmethoxycarbonyl (Fmoc) noncanonical amino acids (ncAAs) enable rational side-chain design to enhance peptide hydrophilicity derived from solid-phase peptide synthesis (SPPS). This review discusses four major classes of ncAAs, cationic, anionic, polar nonionic, and zwitterionic, each improving water solubility through mechanisms such as charge introduction, charge balancing, and strong hydration. Together, these advances demonstrate how ncAAs can be strategically integrated through side-chain engineering in SPPS to produce more water-soluble peptides. Challenges, including side-chain stability and steric hindrance, as well as the need for efficient postassembly conjugation strategies, highlight the need for continued molecular and synthetic innovation, creating opportunities to integrate Fmoc-ncAAs into synthetic peptides for next-generation soluble peptidic biomaterials with broad biomedical impact.

## Graphical abstract

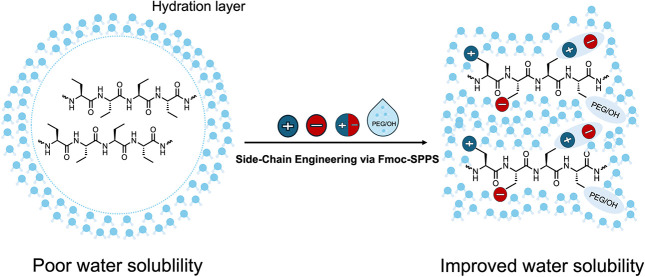

## 1 Introduction

Amino acids (AAs) link through peptide bonds to form peptides, with their side chains largely determining the resulting molecules’ biochemical properties and functions.^1–5^ While the polar peptide backbone (i.e., amide bonds) contributes to aqueous solubility, many synthetic peptides remain poorly soluble due to intramolecular hydrogen-bonding networks and, importantly, the clustering of hydrophobic side chains.^6–8^ Enhancing solubility through side-chain modification is therefore critical for optimizing peptide performance in both biomaterial and biomedical contexts.^2,3,9–11^ This is particularly important for peptidic therapeutics, including glucagon-like peptide-1 receptor agonists and vaccine epitopes, as well as engineered systems such as antibody–drug conjugates,^12^ proteolysis-targeting chimeras, and antimicrobial materials.^13–22^ Hydrophilic modifications similarly improve the biodistribution and diagnostic efficacy of peptide-based imaging agents.^19^ Collectively, these examples underscore that side-chain-driven water solubility is a fundamental design principle, supporting a broad spectrum of biomedical applications.

Current strategies to improve the aqueous solubility of AA-based peptide materials include but not limit to side-chain engineering, backbone polar modification, and control of secondary structural folding.^8^ In this review, we specifically focus on advances in enhancing peptide solubility through side-chain engineering and *in vitro* chemical synthesis approaches, while excluding other strategies (e.g., genetic encoding, biosynthesis, backbone conjugation, folding).^23–25^ Among the chemical synthetic methods, 9-fluorenylmethoxycarbonyl (Fmoc)-based solid-phase peptide synthesis (SPPS) is widely established as the method of choice for *in vitro* preparation.^26–28^ Building on Merrifield’s 1963 method,^29^ SPPS offers several key advantages, including high purity and yield, adaptability to automated and scaled synthesis, and the flexibility to incorporate noncanonical amino acids (ncAAs) and versatile protecting groups.^20^ Importantly, incorporating ncAAs is highly valuable for designing versatile peptide materials, offering a wide array of water-soluble side-chain functionalities. Here, we refer to the water solubility of the peptide after synthesis rather than during the SPPS process. Indeed, water-soluble side chains can be readily incorporated into synthetic peptide materials using both natural and ncAAs. Natural residues bearing charged or polar side chains, including glutamine, asparagine, threonine, serine, glutamic acid, aspartic acid, histidine, lysine, and arginine, have traditionally been exploited to enhance solubility.^2,4,30,31^ In addition, ncAAs with diverse side chains have steadily expanded the repertoire of water-soluble peptide materials. However, comprehensive reviews summarizing ncAAs that enhance water solubility in synthetic peptide-based biomaterials are lacking; although many studies have been reported, they are dispersed across the literature despite strong interest in their biomedical applications.^32–35^

This review is structured to provide a clear chemical framework for water solubility-enhancing amino acid alternatives used in Fmoc-SPPS. Section 2 briefly introduces the fundamental principles of Fmoc-based SPPS. Section 3 discusses natural amino acids and positively charged ncAAs relevant to water solubility, including lysine derivatives, guanidinium-containing residues, imidazolium analogues, and sulfonium based side chain. Section 4 focuses on negatively charged ncAAs such as aspartic/glutamic acid side chains, phosphorylated monomers, and sulfonated analogues. Sections 5 and 6 cover zwitterionic and polar nonionic side chains, respectively—two emerging classes of water-solubilizing motifs. For each section, representative examples of ncAAs and their synthetic routes are provided. To visualize this classification and the structural conventions used herein, a comprehensive overview of these ncAA categories and their carbon nomenclature is presented in Figure 1A–D. Section 7 and 8 address the critical limitations and emerging research directions for water-solubility-enhancing Fmoc-ncAAs. We hope this review serves as a foundational resource in *in vitro* peptide synthesis, highlighting Fmoc-ncAAs that drive the ongoing development of water-soluble peptides for broad biomedical applications.

**Figure 1:**
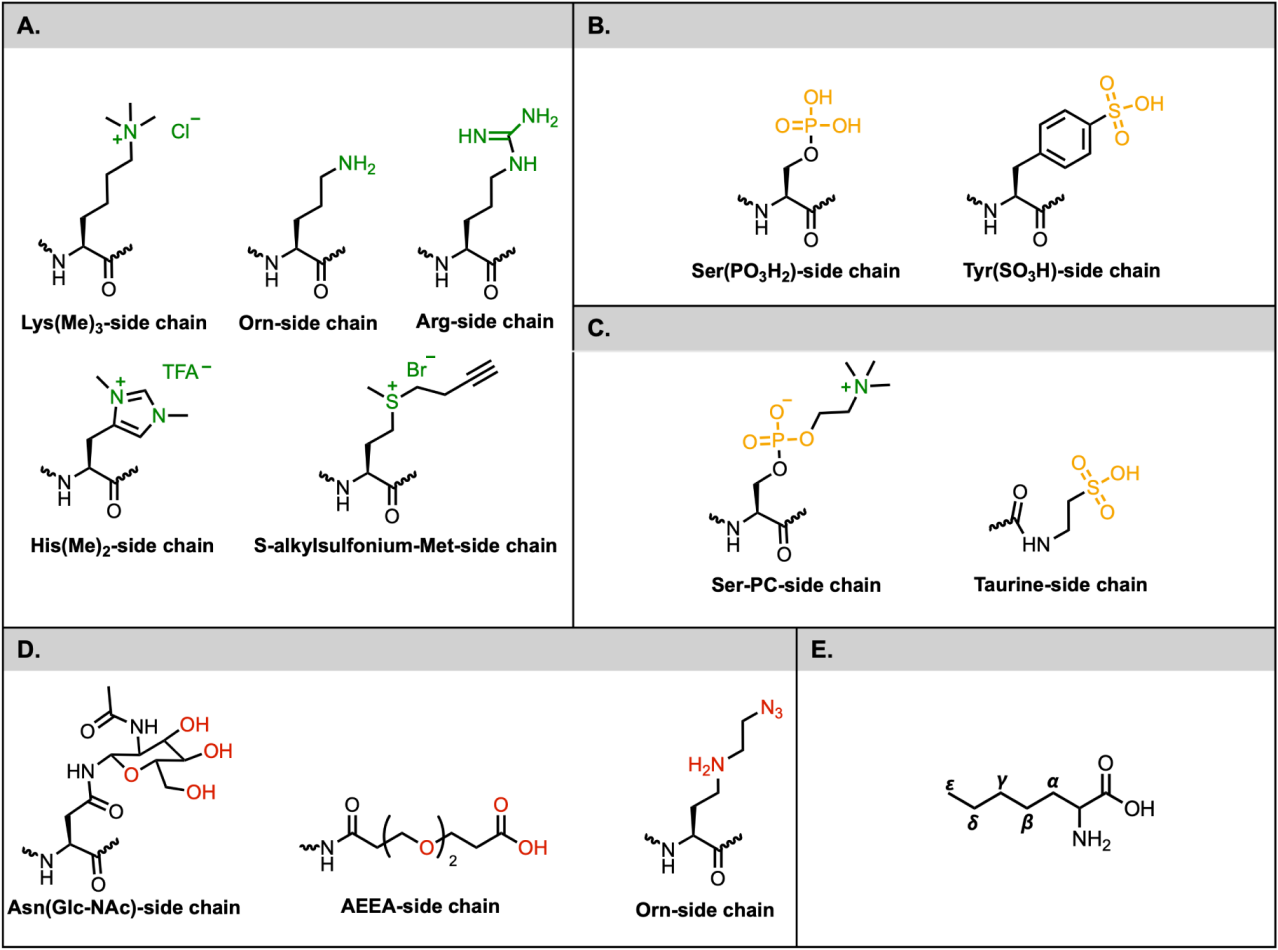
Representative strategies of side-chain engineering to enhance peptide solubility. (A) Positively charged ncAAs: Lys­(Me)_3_, Orn, Arg, His­(Me)_2_, and S-alkylsulfonium-met side chains. (B) Negatively charged ncAAs: Ser­(PO_3_H_2_) and Tyr­(SO_3_H) side chains. (C) Zwitterionic ncAAs: Ser-PC and Taurine side chains. (D) Polar nonionic ncAAs: Asn­(Glc-NAc), AEEA, and Orn side chains. (E) Amino acid carbon nomenclature: Standard α, β, γ, δ, and ε carbon numbering for amino acid side chains. ncAAs = noncanonical amino acids; PC = Phosphorylcholine; AEEA = 2-[(2-Aminoethoxy)­ethoxy] acetic acid.

## 2 Principles of Fmoc-Based SPPS

SPPS, introduced by Merrifield in 1963,^29^ revolutionized peptide chemistry by enabling stepwise elongation of peptides *in vitro* on an insoluble polymer support, simplifying purification through washing and filtration. Among the protection strategies developed, the Fmoc/*tert*-butyl­(tBu) system, established in the 1970s, represented a major improvement over earlier Boc-based methods.^28^ As illustrated in Scheme 1,^27^ the standard workflow for this Fmoc-based strategy involves sequential chain elongation via standard deprotection-coupling protocols, ultimately concluding with concomitant global deprotection and cleavage.

**Scheme 1:**
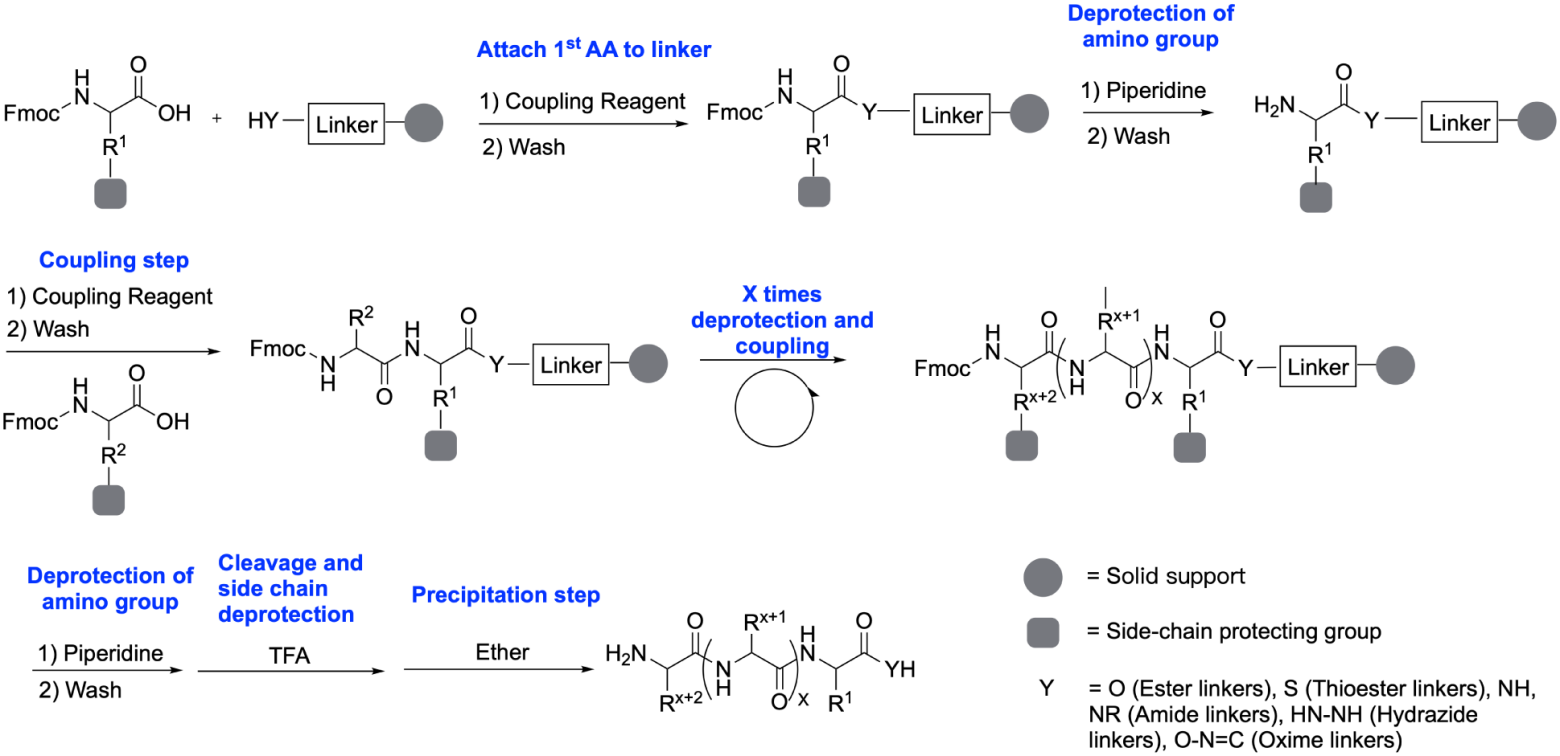
Scheme of Standard Steps in Fmoc SPPS Strategy^a^

a DMF is the primary solvent. Fmoc= 9-fluorenylmethoxycarbonyl, TFA= trifluoroacetic acid, AA= amino acid.^27^ Reproduced from ref 27. Copyright 2017 Springer Nature.

The Fmoc moiety, being sensitive to weak bases, is readily cleaved under mild conditions (typically 20% piperidine in dimethylformamide, DMF). In contrast, tBu-type side-chain protecting groups remain intact until global acid cleavage with trifluoroacetic acid (TFA) is performed.^36^ This orthogonal protection eliminates the use of harsh reagents and ensures compatibility with acid-sensitive functionalities and complex sequences, establishing Fmoc-SPPS as the predominant method in both academia and industry.^28,37^ The Fmoc group is typically introduced using Fmoc-Cl or Fmoc-OSu, and deprotection is monitored by the UV-active dibenzofulvene byproduct.^38^

Effective side-chain protection for amino acids is critical in Fmoc-SPPS to ensure chemoselectivity and prevent side reactions. Standard acid-labile protecting groups are typically employed for canonical amino acids. Specifically, acidic (Asp, Glu) and hydroxyl-containing (Ser, Thr, Tyr) residues are protected as *tert*-butyl esters (−OtBu) and ethers (−tBu), respectively, to prevent unwanted acylation and esterification. Note that the acetinide group is preferentially used for protecting Dopa residues in Fmoc-ncAAs. Basic residues require masking of their nucleophilic amines; typical strategies involve protecting His with trityl (Trt) and Lys with Boc, while Arg is commonly stabilized by the 2,2,4,6,7-pentamethyldihydrobenzofuran-5-sulfonyl (Pbf) group.^39^ Similarly, thiol-containing Cys residues are typically stabilized with Trt groups to prevent oxidation, while aromatic Trp residues are often Boc-protected to suppress indole degradation.^39^ Beyond standard protection, orthogonal protecting groups such as allyloxycarbonyl (Alloc), 4-methyltrityl (Mtt), and 1-(4,4-dimethyl-2,6-dioxocyclohexylidene)­ethyl (Dde) are frequently employed to enable site-selective on-resin modifications.^36^ In contrast, stable functional motifs like azides, alkynes, or zwitterionic groups can often be incorporated without additional protection, provided they tolerate standard Fmoc deprotection and cleavage conditions.^40–43^

Peptide elongation proceeds through coupling reagents such as O-(7-azabenzotriazol-1-yl)-*N*,*N*,*N*′,*N*′-tetramethyluronium hexafluorophosphate (HATU) or *N*,*N*′-diisopropylcarbodiimide (DIC), forming an O-acylisourea or uronium intermediate, respectively.^27,44^ Ethyl cyanohydroxyiminoacetate (Oxyma) or 1-hydroxybenzotriazole (HOBt) is commonly added to suppress racemization and enhance acyl transfer efficiency.^45^ The liberated proton from the amino component is neutralized by a tertiary amine base such as N,N-diisopropylethylamine (DIEA), ensuring complete deprotonation and facilitating nucleophilic attack during amide bond formation.^27,45^ These activation systems are fully compatible with both natural and ncAAs.^27,46^ Importantly, side-chain functionalities such as alkynes, fluorophores, or solubilizing groups can be introduced prior to Fmoc tagging or selectively deprotected on-resin for further conjugation.^47^ These flexible strategies allow for fine control over peptide composition, functionality, and solubility.

In addition, the choice of resin critically influences coupling efficiency, peptide yield, and final product purity in SPPS. For example, provide mechanical stability, while PEG-grafted supports (e.g., ChemMatrix) enhance solvation and reduce aggregation, particularly for long or hydrophobic sequences.^27^ Likewise, linker chemistry defines the C-terminal functionality: Wang resin and CTC resin yield C-terminal carboxylic acids, Rink Amide resin yields C-terminal amides, and safety-catch linkers allow orthogonal or mild cleavage, which is especially useful for fragment condensation or hybrid synthetic strategies.^48^ New resins that can release peptides under water, weak acid, neutral pH conditions, or external stimuli triggers are highly desired and actively being developed.^49–51^

With the advent of automation, SPPS has evolved into a highly efficient and reproducible process. Automated synthesizers precisely control iterative cycles of deprotection, coupling, and washing, thereby significantly improving reproducibility and throughput.^27,52^ Furthermore, microwave-assisted SPPS accelerates both coupling and deprotection steps, reducing reaction times from hours to minutes and improving crude peptide purity, even for sterically hindered or aggregation-prone sequences.^46^

Solvent compatibility is also fundamental to successful SPPS. The standard solvent utilized in Fmoc-SPPS is DMF, often complemented by *N*-methyl-2-pyrrolidone (NMP) for difficult sequences.^53^ Recently, there has been a significant push toward greener alternatives to replace these reprotoxic solvents, including dimethyl sulfoxide (DMSO), 2-methyltetrahydrofuran (2-MeTHF), valerolactone (GVL), and ethyl acetate (EtOAc).^51,53^ The compatibility of developed Fmoc-ncAAs with these solvent systems is a primary prerequisite for ncAAs use in the automated synthesizers (see Section 7.1).

To this end, the versatility of Fmoc-based SPPS, orthogonal protection strategy, and mild reaction conditions permit the incorporation of ncAAs bearing polar, ionic, or zwitterionic side chains, which can markedly enhance peptide solubility and reduce aggregation in water without compromising coupling efficiency or reaction compatibility.^36,52,54^ However, given that DMF serves as the primary solvent in standard SPPS, the solubility and stability of Fmoc-ncAAs in this medium are critical prerequisites, necessitating the use of alternative solvent systems for incompatible monomers. Overall, SPPS provides a robust platform for exploring Fmoc-ncAAs to modulate the solubility of synthetic peptides.

## 3 Fmoc-AAs with Positively Charged Side Chains

When considering solubility-enhancing side chains, the isoelectric point (pI) of a single amino acid may not be very useful because forming the peptide backbone removes its original α-ammonium and α-carboxylate groups. Side-chain properties, such as p*K*_a_ and LogD (Table 1), better predict solubility by reflecting protonation and hydrophobicity after SPPS and under physiological conditions. It is important to acknowledge that the peptide’s N- and C-termini do exert a profound influence on overall solubility by dictating the net charge and pI in a pH-dependent manner.^8^ However, to isolate the specific contributions of residue modifications, this review focuses exclusively on charges derived from side-chain engineering, excluding those arising from the peptide termini.

**Table 1:** Theoretical Physicochemical Properties (Side-Chain pKa and LogD) of Representative Solubility-Enhancing Amino Acid Residues^a^

Category	Amino Acid Name	Symbol	Side-Chain Functional Group	Side-Chain pKa	LogD at pH 7.4 (Hydrophilicity)
Natural Basis	Lysine	Lys	Primary Amine	10.20	–4.08
	Arginine	Arg	Guanidinium	11.66	–4.72
	Histidine	His	Imidazole	13.8	–1.93
	Aspartic Acid	Asp	Carboxylate	4.20	–5.02
Cationic ncAAs	Homoarginine	Agh/Har	Guanidinium (Longer linker)	12.06	–3.83
	Ornithine	Orn	Primary Amine (−1 CH_2_)	9.60	–4.07
	Diaminobutyric acid	Dab	Primary Amine (−2 CH_2_)	9.77	–4.31
	Diaminopropionic acid	Dap	Primary Amine (−3 CH_2_)	8.09	–3.26
	Trimethyllysine	Lys (Me)_3_	Quaternary Ammonium	-	–4.82
	Dimethylhistidine	His(Me)_2_	Imidazolium	-	–5.57
	S-alkylsulfonium Met	Met(S+)	Sulfonium	9.61	–0.47
Anionic ncAAs	Phosphoserine	pSer	Phosphate (Monoester)	6.39	–5.92
	Phosphothreonine	pThr	Phosphate (Monoester)	6.37	–5.52
	Phosphotyrosine	pTyr	Phosphate (Monoester)	6.75	–3.75
	Sulfotyrosine	Tyr (SO_3_H)	Sulfate/Sulfonate	–1.15	–2.88
Zwitterionic	Ser-Phosphorylcholine	Ser-PC	Phosphorylcholine	-	–4.26
	Peptidosulfonamide		Taurine-like (Sulfonate)	-	–4.16
Polar Nonionic	N-GlcNAc-Asparagine	Asn (Glycan)	Carbohydrate (Polyol)	13.3	–5.27
	Mini-PEG (AEEA)	AEEA	Ether/Amide backbone	4.34	–3.97
	Azido-amine		Secondary Amine + Azide	8.72	–7.29

a All values are estimated for amino acid residues when embedded within a peptide backbone, modeled as *N*-acetyl-l-amino acid-*N*-methylamide (Ac-Xaa-NHMe) to exclude terminal charge effects. Structures are analyzed in their deprotected, physiologically active forms. The p*K*_a_ and LogD (at pH 7.4) values were calculated using MarvinSketch (ChemAxon) to assess ionization and hydrophilicity under physiological conditions, where lower LogD values indicate greater hydrophilicity. In general, a LogD7.4 value below 0 is considered hydrophilic, while values above 0 suggest increased hydrophobicity; notably, a LogD7.4 of ∼1–3 is often considered proper for drug candidates, as it provides a balanced profile between hydrophilicity and lipophilicity. “N/A” indicates the side chain possesses a permanent charge or is nonionizable.

In this subsection, we focus on positively charged side chains that enhance solubility through stable cationic functionalities, including ε-amino based lysine derivatives, guanidinium containing residues, imidazolium analogues, and sulfonium based side chains. These classes collectively illustrate how cationic motifs improve synthetic phase manageability and aqueous solubility through charge density, hydrogen bonding capacity, and electrostatic interactions. It is worth noting that for many amino acids, particularly basic residues such as Lys and Arg, systematic homologous series also exist, in which the side chain carbon length is extended by +1, +2, or +3 carbons (e.g., homo-, homohomo-, and trihomo-analogues such as Fmoc-HArg-OH). These homologated derivatives follow similar chemical principles and may alter solubility or charge spacing, although detailed studies remain limited; thus, they are acknowledged here but not discussed extensively.

### 3.1 Lysine Derivatives

Charged amino acids play a central role in modulating peptide solubility, aggregation, and overall physicochemical behavior.^55^ Positively charged residues (e.g., Lys, Arg, His) promote solubility through cation dipole interactions and electrostatic attraction with anionic partners.^56^ Among these, lysine and its derivatives are the most widely explored for improving solubility because of the synthetic versatility of their ε-amino groups.

A representative example is Fmoc-Lys­(Me_3_)–OH, which features a quaternary ammonium group that maintains a permanent positive charge, markedly enhancing peptide solubility in both organic and aqueous media. It is typically synthesized via stepwise methylation of ε-amino-protected lysine derivatives using methyl iodide or methyl triflate under anhydrous conditions, with subsequent installation of the Fmoc group at the Nα-position. This quaternized lysine derivative facilitates the synthesis of highly hydrophobic peptide sequences by improving resin swelling and minimizing aggregation during coupling.^57^

In addition to direct modification, shortening the alkyl spacer of lysine represents another robust strategy. Lysine homologues such as Ornithine (Orn), Diaminobutyric acid (Dab), and Diaminopropionic acid (Dap) introduce primary amino groups with shorter carbon chains (1 to 3 carbons) compared to the 4-carbon chain of lysine. Representative examples include Fmoc-Orn (Boc)–OH, Fmoc-Dab­(Boc)–OH, and Fmoc-Dap (Boc)–OH, which increase hydrogen-bonding capacity and overall charge density while reducing the hydrophobic bulk of the side chain. These residues have been shown to enhance peptide solubility in DMF and aqueous media by reducing backbone folding and suppressing aggregation during chain elongation.^58^ Building upon this concept, orthogonally protected analogues such as Fmoc-Dap (Alloc)–OH and Fmoc-Dab (Mtt)–OH allow for selective on-resin deprotection.^59,60^ This enables postassembly conjugation with hydrophilic groups (e.g., short PEG chains or polar linkers), effectively turning these residues into chemical handles for further solubility tuning and bioconjugation.^60^

### 3.2 Guanidinium Side Chains

The guanidinium functionality is among the most potent cationic motifs in peptide chemistry, owing to its high p*K*_a_ (13.6), charge delocalization, and strong hydrogen-bonding and cation−π interactions.^61^ In Fmoc-based SPPS, the arginine guanidinium group allows interactions in three possible directions with anionic counterparts through its three nitrogen atoms, compared to the single direction offered by the ammonium group of lysine. Consequently, guanidinium-bearing residues enhance solubility and stabilize secondary structures through strong electrostatic pairing with carboxylates.^62^

A systematic examination of β-hairpin peptides containing guanidinium and carboxylate residues revealed the critical role of side-chain length in stability.^63^ Using Fmoc-based synthesis, a homologous series of chain-length variants (Agp, Agb, Arg, and Agh) were incorporated at positions opposite acidic residues (Asp, Glu, Aad). Peptides incorporating preguanidinylated monomers, such as Fmoc-Agh­(Boc)_2_–OH, were assembled directly by standard coupling, while on-resin guanidinylation was achieved for shorter analogues via selective (4-methyltrityl) Mtt removal followed by reaction with di-Boc-triflylguanidine. Shorter analogues required repeated couplings due to steric hindrance near the backbone. Nuclear magnetic resonance (NMR) and thermodynamic analyses revealed that long-chain guanidinium residues (Agh and Arg) yielded greater β-hairpin folding and stability than short-chain analogues (Agp and Agb). Only long donor–acceptor pairs provided measurable stabilizing interactions, indicating that side-chain length matching between guanidinium donors and carboxylate acceptors is critical for optimizing folding and solubility behavior.^63^

Guanidinium modified amino acids remain highly soluble and reactive in DMF/NMP, and the resulting peptides display monomeric behavior over a broad concentration range, minimizing aggregation during synthesis and solution studies. Collectively, guanidinium bearing ncAAs particularly Agh and its Fmoc-protected derivatives, offer a robust route to enhance synthetic phase manageability and aqueous solubility. Their tunable side-chain geometry also provides a rational handle for fine-tuning charge pairing and structural organization in designed β-structures.^63^

### 3.3 Imidazolium-Based Side Chains

Imidazolium-based side chains offer a permanently positively charged heteroaromatic motif that mimics the histidine side chain but with enhanced polarity and insensitivity to pH changes. A representative example is the incorporation of *N*,*N*-dimethylhistidine, which features a quaternary imidazolium ring. The synthesis of the Fmoc-protected analogue, Fmoc-His­(Me)_2_–OH, was achieved starting from commercially available N-Boc-l-histidine via side-chain methylation followed by protecting group exchange.^64^ Interestingly, the introduction of the Fmoc group was found to be chemically sensitive; the imidazolium moiety activated the α-amino group, leading to the formation of a bis-Fmoc species (Nα, Nα-diFmoc) under standard Schotten-Baumann conditions. Despite this unusual reactivity, the resulting imidazolium building block was fully compatible with standard Fmoc-SPPS protocols, allowing for the efficient assembly of linear peptides with high purity. Although originally designed for metal coordination, the introduction of such permanently charged imidazolium residues provides a robust strategy for increasing peptide polarity and modulating solubility through strong cation dipole interactions.^64^

### 3.4 Sulfonium-Based Side Chains

Unlike other cationic residues, sulfonium-based side chains (such as S-methylmethionine analogues) are generally chemically unstable under the repetitive base treatments (e.g., piperidine) required for Fmoc removal. Therefore, they are typically introduced via a postassembly alkylation strategy on standard Fmoc-Methionine residues. A robust methodology to introduce these unstable motifs involves the assembly of peptides via standard Fmoc-SPPS, followed by chemoselective S-alkylation of the methionine side chain (Figure 2A). This conversion transforms the hydrophobic thioether of methionine into a permanently positively charged sulfonium center. This modification not only significantly enhances the aqueous solubility of the peptide due to the high polarity of the ionic sulfonium group but also introduces a reactive handle for further bioorthogonal functionalization. Thus, this strategy effectively expands the toolbox of Fmoc-compatible solubilizing tags by repurposing the natural methionine residue.^65^

**Figure 2:**
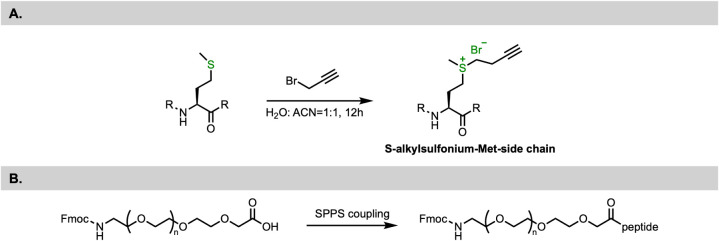
Specific synthetic strategies and challenges for introducing specialized solubilizing groups. (A) Synthesis of S-alkylsulfonium-Met-side chain.^65^ (B) PEGylation strategy.^66^ (A) Reproduced from ref 65. Copyright 2023 Elsevier. (B) Reproduced from ref 66. Copyright 2007 American Chemical Society.

### 3.5 Concluding Remarks

Positively charged side chains improve peptide solubility mainly through electrostatic repulsion and hydration shell formation, which disrupt hydrophobic aggregation during both synthesis and in solution. Quaternary ammonium and guanidinium groups maintain stable positive charges that enhance resin swelling and coupling efficiency in SPPS. These cationic side chains are widely used in cell-penetrating peptides, antimicrobial peptides, biofilm-disrupting peptides, and gene-delivery systems. Future work may focus on designing Fmoc-compatible cationic ncAAs with optimized charge spacing and structural flexibility or combining them with zwitterionic and polar nonionic motifs to achieve balanced hydration and synthetic efficiency.

## 4 Fmoc-AAs with Negatively Charged Side Chains

Negatively charged side chains enhance peptide solubility primarily through electrostatic repulsion and the formation of strongly bound hydration shells. In this section, we focus on three major classes of anionic residues relevant to Fmoc-based SPPS: (i) the natural carboxylate-bearing amino acids Asp and Glu, (ii) phosphorylated side chains that introduce dense ion-dipole interactions, and (iii) sulfonated or sulfated analogues that provide permanent, hydrolytically stable negative charges. Together, these motifs illustrate the breadth of anionic designs available for modulating solubility and synthetic behavior in peptide systems.

### 4.1 Aspartic and Glutamic Acid Side Chains

Aspartic acid and glutamic acid are the natural anionic residues in SPPS method, characterized by side-chain carboxylates with p*K*_a_ values of 4.07 and 3.90, respectively, and are introduced as Fmoc-Glu (OtBu)–OH and Fmoc-Asp (OtBu)–OH.^38,67^ The tBu groups protect the side-chain carboxylates from unwanted acylation and intramolecular cyclization (e.g., aspartimide formation) during peptide assembly, and are cleaved during global TFA treatment.^68,69^ Upon deprotection, the resulting free carboxylates (−COO^–^) enhance solubility by engaging in strong ion–dipole interactions with water and acting as hydrogen bond acceptors to form a hydration shell. Furthermore, the introduction of negative charges creates electrostatic repulsion between peptide chains, effectively inhibiting hydrophobic aggregation. A major synthetic challenge for these residues is aspartimide formation, particularly in Asp–Gly and Asp–Asn motifs. Several strategies have been developed to suppress this side reaction.^68,69^ Albericio and coworkers^53^ demonstrated that shortening deprotection times and lowering temperatures effectively minimize cyclization. Backbone amide protection using hydroxymethylnitrobenzyl or side-chain protection via 4-(dimethylamino)­benzyl can also prevent intramolecular ring formation.^53,70^ More recently, cyanosulfurylide masking at the β-carboxylate has shown excellent aspartimide suppression while maintaining Fmoc-SPPS compatibility.^71,72^

### 4.2 Phosphorylated Side Chains

Phosphorylated amino acids are among the most extensively studied anionic ncAAs, valued for enhancing peptide solubility and mimicking biological phosphorylation. The phosphate group acts as a dibasic acid with p*K*_a1_ = 2.14 and p*K*_a2_ = 7.20. Consequently, at physiological pH, it exists primarily as a dianion (-PO_3_^2–^). This high charge density significantly increases hydrophilicity via strong ion–dipole interactions and electrostatic repulsion, which disrupt hydrophobic aggregation. Two main synthetic routes have been employed in Fmoc-SPPS: postassembly phosphorylation and preformed phosphorylated monomers.^24^

Postassembly phosphorylation: The fully assembled peptide containing unmodified hydroxyl residues (Ser, Thr, or Tyr) is treated with phosphitylating reagents such as Cl–P­(O)­(OR)_2_ or P­(OCH_2_CH_2_CN)­(OR)_2_ in the presence of *N*-methylimidazole or DIEA, followed by oxidation (I_2_ or tBuOOH). This approach allows late-stage modification with minimal steric hindrance, though efficiency depends on sequence and protecting-group stability.^24^

Prephosphorylated building blocks: Monomers such as Fmoc-Ser­(PO­(OBzl)_2_)–OH, Fmoc-Thr­(PO­(OBzl)_2_)–OH, and Fmoc-Tyr­(PO­(OBzl)_2_)–OH have been widely used. Protecting groups including Bzl, tBu, or Pac confer base stability during Fmoc removal and are cleanly removed in TFA. Efficient coupling of these monomers has been demonstrated under HATU/DIEA or DIC/Oxyma conditions, yielding high site selectivity and reproducibility.^73^

Protecting-group selection strongly influences yield and stability: Bzl-protected phosphates provide higher base stability, whereas tBu analogues simplify deprotection but are less robust for long sequences. Postcoupling oxidation and neutralization, typically with NH_4_HCO_3_ or NaOAc, prevent phosphate migration and preserve product integrity. Together, these strategies enable precise control of phosphorylation sites, offering both synthetic versatility and improved solubility for peptide assemblies. However, a significant limitation of phosphorylated peptides is their susceptibility to rapid hydrolysis by endogenous phosphatases, which can lead to premature dephosphorylation and loss of the solubility enhancing modification.^74^

### 4.3 Sulfonated and Sulfated Side Chains

Sulfonated and sulfated amino acids represent another important class of negatively charged residues that permanently increase polarity and mimic biological motifs such as sulfotyrosine and sulfoserine. Unlike carboxylates or phosphates, these functional groups behave as strong acids with extremely low p*K*_a_ values (typically <1). Consequently, they remain fully deprotonated across the entire physiological pH range and even under acidic conditions. The sulfonate group imparts a strong, permanent anionic character that enhances solubility and suppresses hydrophobic aggregation.^23,75^

On-resin sulfation: As outlined in Scheme 2A, Kiessling et al. demonstrated that Tyr (OAzm) derivatives can be selectively sulfated on-resin using DMF·SO_3_ complexes under mild and Fmoc-compatible conditions. The resulting Tyr (SO_3_H) containing peptides exhibited high solubility and structural integrity after TFA cleavage.^23^

**Scheme 2:**
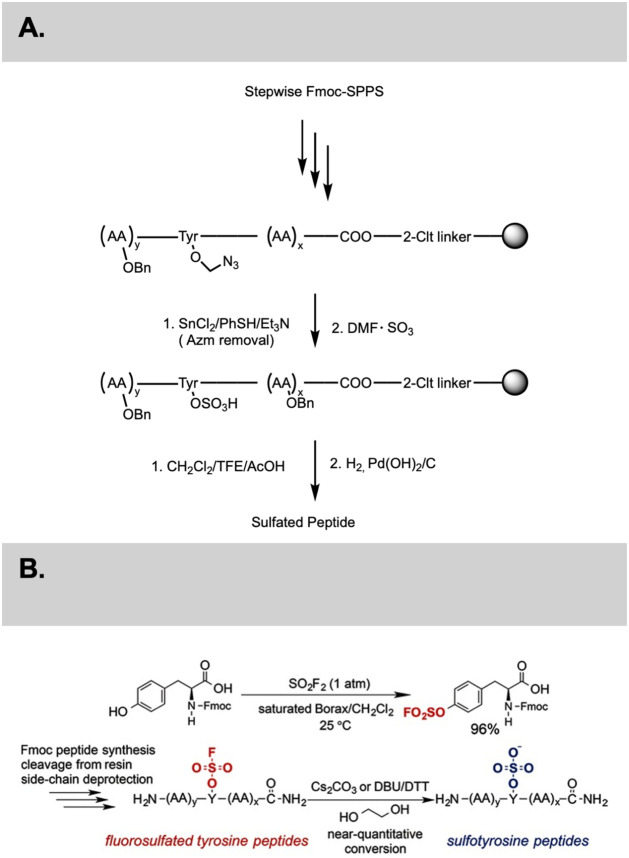
(A) Overview of sulfated peptide synthesis;^23^ (B) Overview of the synthesis of sulfotyrosine peptides;^75^ (A) Reproduced from ref 23. Copyright 2002 John Wiley and Sons; (B) Copyright 2015 John Wiley and Sons

Sulfur–fluoride exchange (SuFEx) fluorosulfate chemistry: Sharpless and coworkers later developed a SuFEx route, converting Tyr­(OSO_2_F) residues into Tyr­(OSO_3_H) under mildly basic aqueous conditions (Na_2_CO_3_ or tertiary amines) as illustrated in Scheme 2B. This reaction provides better control of sulfation stoichiometry and avoids oversulfation.^75^ Expanding the utility of this chemistry, a tyrosine-selective macrocyclization strategy was recently reported to access sulfonate-tyrosine ester macrocycles, termed STEMtides.^76^ This approach leverages SuFEx to target tyrosine phenol moieties using sulfonyl fluoride electrophiles in aqueous buffer under mild conditions, achieving chemoselective cyclization without additional reagents. The method demonstrates high tolerance for native side chains and has been successfully applied to synthesize biologically active analogs of clinically relevant peptides, such as leuprorelin and cilengitide, highlighting the translational potential of these new peptide macrocycles.^76^

Dichlorovinyl (DCV) protection strategy: Taylor and coworkers^77^ established an efficient protocol by incorporating sulfotyrosine as a DCV-protected diester to mask the labile sulfate group. To prevent sulfate elimination during chain assembly, 2-methylpiperidine was employed for Fmoc removal instead of piperidine. The final DCV protecting group was cleaved via mild hydrogenolysis, delivering sulfotyrosine peptides in good yield while avoiding desulfation.^77^

Both sulfation routes are compatible with Fmoc-SPPS and afford permanently charged peptides with strong hydration shells and electrostatic repulsion, leading to excellent solubility and purification behavior. Unlike phosphorylated analogues, sulfonate groups are chemically stable and resist hydrolysis during TFA cleavage.^23,75^

### 4.4 Concluding Remarks

Negatively charged residues such as phosphorylated or sulfonated analogues and positively charged residues particularly guanidinium and sulfonium derivatives, share a fundamental solubility-enhancing mechanism: both generate dense hydration shells and induce electrostatic repulsion that minimizes peptide–peptide association in organic and aqueous environments. Beyond this commonality, cationic residues are particularly effective at disrupting -sheet aggregation by interfering with backbone hydrogen bonding. Regarding stability, anionic residues generally excel in improving aqueous behavior near neutral pH, whereas cationic residues offer robust stability under acidic cleavage conditions. Reflecting these properties, anionic motifs are widely employed in anticoagulant peptides, wound healing, and biocompatible drug delivery systems. While these charged motifs rely on long-range electrostatic interactions, the zwitterionic and polar nonionic modifications discussed in the next section achieve solubility through charge neutrality and extensive hydration.

## 5 Fmoc-AAs with Zwitterionic Side Chains

Zwitterionic side-chain modification has emerged as an effective approach to restore charge balance and hydration capacity in peptides.^78^ Compared with the above charged residues, zwitterionic motifs could provide superior solubility and antifouling performance, particularly under physiological or high-ionic-strength conditions.^25^ It can be misleading to call peptides zwitterionic (as AAs can be at certain pH) because peptide bonds remove the α-ammonium and α-carboxylate groups; in this review, “zwitterionic” refers only to side-chain groups. Models and structures of zwitterionic materials as shown in Figure 3A–C.^25^ Indeed, adding both positive and negative groups to a side chain keeps internal charge balance, creates strong hydration shells, and prevents unwanted aggregation.^79^

**Figure 3:**
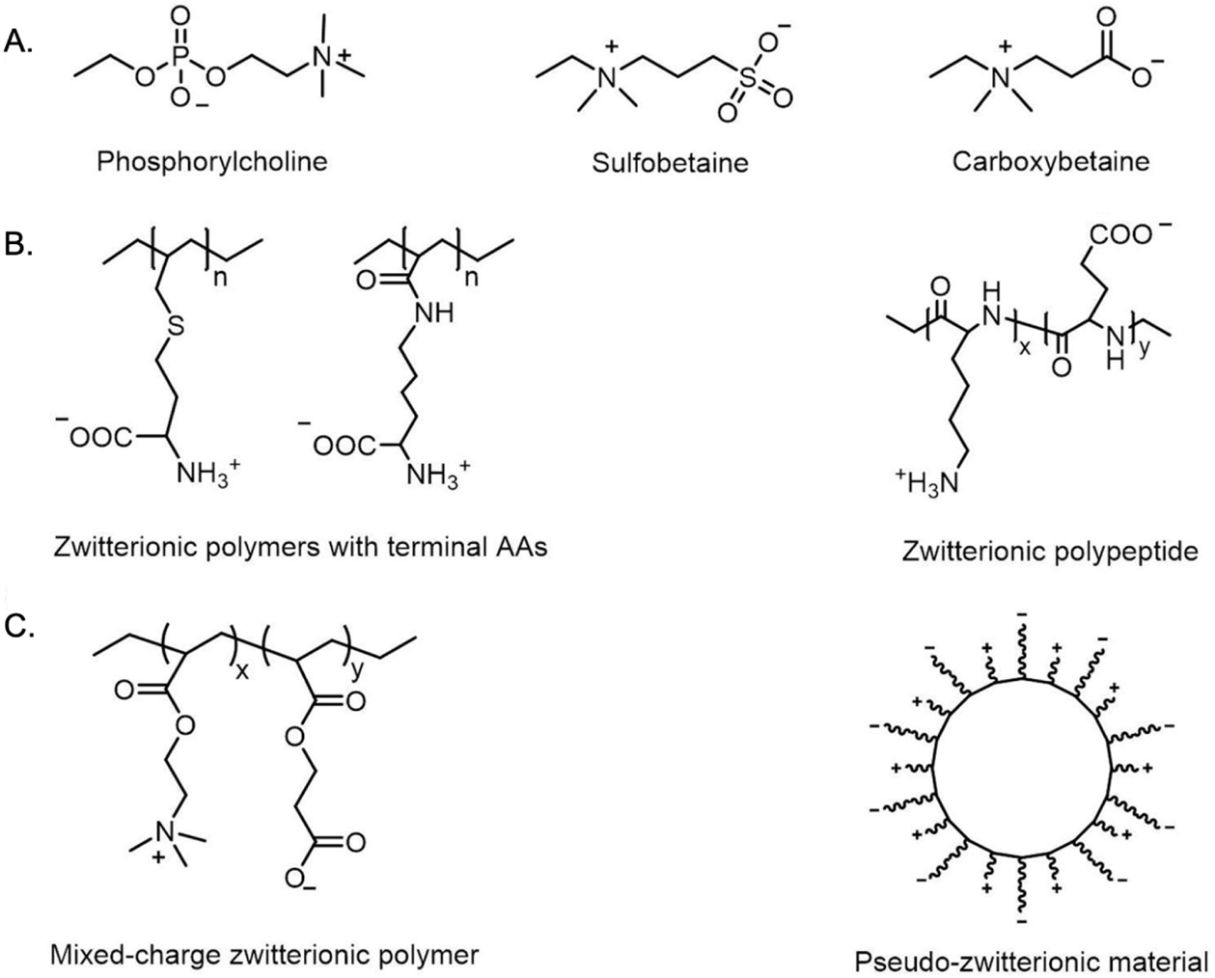
Structural classification and molecular architectures of zwitterionic materials. (A). Chemical structures of representative zwitterionic moieties commonly used in biomaterials. (B). Zwitterionic poly­(amino acids) and polypeptides incorporating intrinsic zwitterionic side chains. (C). Mixed-charge systems achieving overall neutrality, including polyampholytes composed of balanced cationic and anionic monomers, and pseudozwitterionic assemblies formed via stoichiometric electrostatic binding.^25^ Copyright 2020 Elsevier.

### 5.1 Phosphorylcholine Side Chains

Phosphorylcholine (PC)–modified amino acids represent the most established class of zwitterionic residues compatible with Fmoc-SPPS. The phosphorylcholine headgroup, containing both a quaternary ammonium and a phosphate anion, mimics the hydrophilic moiety of natural phospholipids.^78^ As illustrated in Scheme 3, Albers and Hedberg developed Fmoc-Ser­(PO–OCH_2_CH_2_N^+^ (CH_3_)_3_), Fmoc-Thr­(PO–OCH_2_CH_2_N^+^ (CH_3_)_3_), and Fmoc-Tyr­(PO–OCH_2_CH_2_N^+^ (CH_3_)_3_) derivatives by coupling a phosphoramidite intermediate to Nα-Fmoc-protected allyl esters of hydroxyl amino acids, followed by oxidation and allyl deprotection. The resulting PC-functionalized monomers were introduced into peptides using standard HATU/DIEA couplings without side reactions or phosphate loss. Peptides incorporating phosphorylcholine side chains showed markedly enhanced aqueous solubility and strong antifouling behavior. The zwitterionic PC group preserved its charge-neutral structure through all Fmoc deprotection and TFA cleavage steps, confirming full synthetic compatibility. These results established phosphorylcholine as a reliable model for designing zwitterionic ncAAs in Fmoc-based peptide synthesis.^73^

**Scheme 3:**
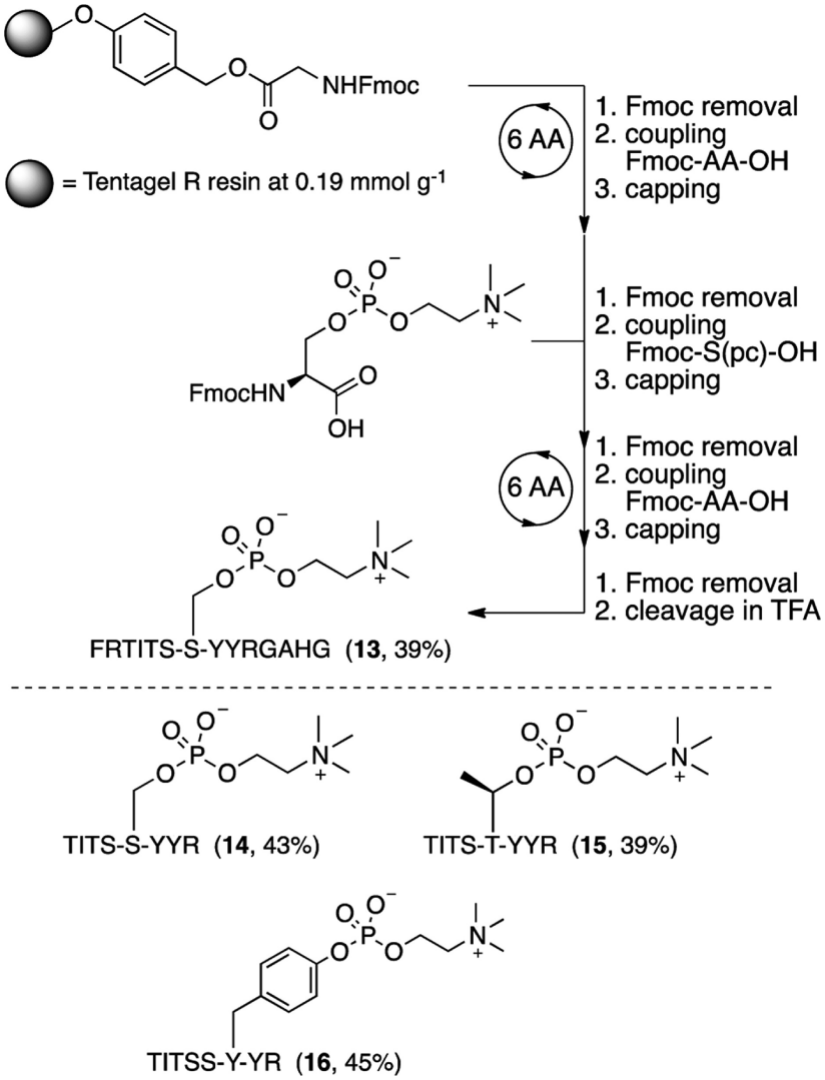
Fmoc Solid-Phase Peptide Synthesis of Phosphocholinated Peptides^73^ [Fn sch3-fn2]

a Reproduced from ref 73. Copyright 2013 American Chemical Society.

### 5.2 Taurine Side Chains

Another class of zwitterionic residues originates from sulfonate–ammonium pairing, exemplified by taurine-based amino acids. Taurine (2-aminoethanesulfonic acid) naturally carries both a sulfonate and a protonated amine, creating an intrinsic internal charge balance. The incorporation of taurine-like residues has been achieved through benzotriazole-mediated coupling, producing peptides with terminal sulfonate groups.^80^ Furthermore, Fmoc-compatible sulfonate chemistry allows for the partial replacement of amide bonds with sulfonamide linkages using activated aminoethanesulfonyl chlorides, yielding analogues that remain stable under repeated base-deprotection cycles.^81^

These activated monomers were incorporated into peptides via Fmoc-SPPS on Rink or Wang resins, partially replacing amide bonds with sulfonamide linkages. The resulting peptidosulfonamide analogues were obtained in high yields and purity, remaining stable under repeated base-deprotection cycles. Functionally, C-terminal sulfonamide analogues retained comparable binding activity to native Leu-enkephalin, demonstrating both chemical robustness and functional integrity. Collectively, these examples illustrate the feasibility of integrating sulfonate-ammonium-type zwitterionic motifs within Fmoc workflows without compromising synthetic reliability.^80,81^

### 5.3 Concluding Remarks

Zwitterionic side chains, particularly phosphorylcholine- and taurine-based motifs, significantly improve solubility and chemical stability in Fmoc-SPPS-derived peptides. Their intrinsic charge neutrality and strong hydration enable the design of nonaggregating, biocompatible sequences suitable for biomedical applications.^82^ Although individual zwitterionic molecules are overall charge-neutral, the effective charge of zwitterionic peptide assemblies is not necessarily neutral, being governed largely by the spatial arrangement, charge seperation, and polarizability of the zwitterionic groups. The spatial distribution, inter-charge spacer, and alignment of positive and negative moieties at the assembly interface govern how peptides interact with surrounding water molecules, thereby influencing hydration behavior and the resulting effective surface charge of the assembled structure.

Crucially, this dense and tightly bound hydration layer serves as a physical barrier that endows zwitterionic peptides with exceptional antifouling properties. By effectively preventing nonspecific protein adsorption and cell adhesion, these motifs offer a superior alternative to traditional PEGylation, avoiding issues such as oxidative degradation and the induction of anti-PEG antibodies (the ABC phenomenon). Consequently, zwitterion-functionalized peptides are increasingly recognized for their “stealth” behavior, extending circulation half-life in drug delivery applications.

Although other systems, such as carboxybetaine, sulfobetaine, sulfabetaine, and imidazolium propionate analogues, have yet to be validated under Fmoc conditions, their success in polymer and biomaterial chemistry suggests strong potential for peptide synthesis. Future work should focus on developing orthogonally protected zwitterionic building blocks that retain hydration capacity and structural integrity through the entire SPPS workflow, extending the solubility-enhancing advantages of zwitterions to broader peptide systems.

## 6 Fmoc-AAs with Polar Nonionic Side Chains

Polar nonionic side chains enhance peptide solubility without introducing a net charge, offering a versatile strategy to mitigate aggregation and improve handling during Fmoc-SPPS. Natural polar residues such as Ser, Thr, Asn, and Gln provide limited polarity and often fail to prevent precipitation in hydrophobic sequences. To address this limitation, synthetic amino acids bearing polar yet charge-neutral functionalities, such as sugar-derived and azide-containing side chains, have been developed to improve solvent compatibility while maintaining synthetic robustness under Fmoc conditions. In this section, we briefly highlight representative classes of polar nonionic side chains, primarily sugar-derived and azide-containing amino acids, as each subclass comprises relatively concise but illustrative examples of charge-neutral strategies for enhancing peptide solubility. Although individually short, these cases collectively demonstrate the breadth of polar functionalities that remain compatible with Fmoc-SPPS and provide effective solubility enhancement.

### 6.1 Sugar-Derived Side Chains

Carbohydrate-modified residues introduce multiple hydroxyl groups that form extended hydration shells, markedly enhancing solubility. Sugar-based amino acids include β-sugar analogues, O-glycosylated Ser/Thr/Tyr derivatives, and N-glycosylated Asn variants; S-glycosylation serves as a more acid/base-stable alternative within Fmoc workflows.^83–89^ Detailed discussions on these glycosylation chemistries can be found in a recent comprehensive review.^90^

#### 6.1.1 β-Sugar Amino Acids

Representative Fmoc-β-sugar monomers such as Fmoc-GlcAPC­(Ac)–OH are synthesized from per-acetylated d-glucosamine via TMSCN/BF_3_·OEt_2_ cyanation, hydrolysis, and Fmoc protection. Their O-acetyl groups remain intact through 20–40% piperidine deprotection and TFA cleavage, enabling final Zemplén deacetylation to afford hydroxyl-rich, highly soluble peptides. Couplings on Tentagel RAM resin with PyBOP/DIEA achieve >80% efficiency, and the resulting products exhibit excellent solubility in aqueous or mixed organic media.^88^

#### 6.1.2 O-Glycosylated Amino Acids

Nakahara et al. synthesized core-3 and core-6 O-glycan-linked glycopeptides by coupling benzyl-protected Ser/Thr-based glycoamino acids onto Fmoc-CLEAR resin using HBTU/HOBt activation, followed by mild TfOH-mediated global debenzylation. These peptides, derived from MUC2 and MUC6 mucin fragments, exhibited high synthetic yields and retained glycan integrity through multiple deprotection cycles. Subsequent enzymatic sialylation confirmed the compatibility of these glycopeptides with postsynthetic enzymatic modification, highlighting the robust behavior of O-glycosylated residues under Fmoc conditions.^85,89^ Similarly, Kasteren and coworkers incorporated benzyl-protected disaccharides into MUC1-derived 20-mer sequences using HBTU/HOBt coupling at 50 °C and subsequently removed benzyl protections with TfOH/soft nucleophiles. The resulting *O*-glycopeptides showed enhanced solubility and structural fidelity during Fmoc-SPPS.^83^

#### 6.1.3 S-Glycosylated Amino Acids

While natural *O*-linked glycosylation is a highly effective strategy for improving peptide solubility, it suffers from inherent synthetic and biological limitations.^91^ Notably, *O*-glycosidic bonds are susceptible to β-elimination under the standard basic deprotection conditions of Fmoc-SPPS (e.g., repeated piperidine treatments) and are prone to rapid enzymatic hydrolysis by native glycosidases *in vivo*. To overcome these challenges, *S*-linked glycosylation has emerged as a robust bioisosteric alternative.^92^ A comprehensive comparison of the physicochemical properties and synthetic utility between native *O*-linked and bioisosteric *S*-linked glycosyl amino acids is summarized in Table 2.

**Table 2:** Comparison of Physicochemical Properties and Synthetic Utility between Native *O*-Linked and Bioisosteric *S*-Linked Glycosyl Amino Acids

Feature	O-Glycosylation (Native)	S-Glycosylation (Bioisostere)
Chemical Stability (SPPS)	Susceptible to β-elimination. Under basic conditions (e.g., piperidine treatment), the glycan moiety can be cleaved, requiring careful optimization.	Chemically robust. The C–S bond is highly stable against acids and bases, resistant to β-elimination, and fully compatible with standard Fmoc-SPPS protocols.
Enzymatic Stability (In Vivo)	Labile. Readily hydrolyzed by native glycosidases and esterases in biological fluids, limiting serum half-life.	Highly Stable. Resistant to enzymatic hydrolysis due to the non-native sulfur linkage, significantly prolonging biological half-life.
Synthetic Accessibility	Moderate to Difficult. Stereoselective synthesis of glycosidic bonds is complex; building blocks are often expensive or require elaborate protection strategies.	Good. S-linked building blocks are readily synthesized via nucleophilic substitution; they are stable intermediates that simplify the SPPS workflow.
Structural Conformation	Native conformation. Dictates the natural folding and molecular recognition of glycoproteins.	Isostructural mimic. Despite a longer C–S bond length (approximately 1.8 Å vs 1.4 Å for C–O), it closely mimics the native conformation and maintains biological activity.
Solubility Enhancement	Excellent. Provides substantial hydrophilicity through multiple hydroxyl groups.	Excellent. Comparable hydrophilicity and hydration capacity to native O-glycans.

By replacing the native oxygen atom with sulfur, *S*-glycosylated amino acids introduce thioglycosidic linkages that resist both acid and base cleavage. To provide streamlined access to these building blocks, a recent methodology takes advantage of the *in situ* generation of glycosylthiolates from carbohydrate acetates. Under mild basic conditions, these reactive intermediates undergo stereoselective conjugation with Fmoc-iodo-amino acids, yielding diverse *S*-glycosylated monomers in high yields. Validating this strategy, these building blocks were successfully employed in standard SPPS, notably enabling the novel integration of extended thio-oligosaccharide chains directly into the peptide chain.^84^ This robust synthetic route highlights the potential of *S*-glycosylation to introduce massive, highly stable solubilizing motifs without compromising the Fmoc-SPPS workflow.

#### 6.1.4 N-Glycosylated Amino Acids

Asn­(glycan) building blocks extend the repertoire of polar nonionic amino acids, combining high hydrophilicity with enzymatic compatibility. These are typically prepared by selective Staudinger reduction of glycosyl azides followed by ring-opening of Fmoc-aspartic anhydride, generating Fmoc-Asn­(glycan)–OH derivatives suitable for automated SPPS. The introduction of Fmoc-Asn­(N–Ac_3_GlcNAz)–OH further integrates a bioorthogonal azide handle, enabling both enhanced solubility and postsynthetic click reactions. Additionally, enzymatic glycan remodeling via ENGase-mediated oxazoline transfer validates the structural stability of these glycoamino acids in solid-phase peptide synthesis.^83^

#### 6.1.5 Key Practical Features

Effective use of sugar-derived side chains relies on stable protecting groups (O–Ac, benzyl), extended coupling times or mild heating (∼50 °C), and optional enzymatic extension after assembly. Collectively, these strategies provide precise control of hydrophilicity, allowing difficult or aggregation-prone peptides to remain soluble throughout synthesis and purification.

### 6.2 Sulfoxide-Containing Side Chains

The oxidation of Met to methionine sulfoxide [Met­(O)] represents an effective strategy for introducing polar, nonionic functionality into peptide sequences.^93^ The native thioether side chain of methionine is intrinsically hydrophobic and often contributes to peptide self-assembly and aggregation through hydrophobic interactions.^94^ Upon oxidation, the formation of a sulfoxide group (>S = O) introduces a strong dipole moment, substantially increasing side-chain polarity and hydration while preserving overall charge neutrality.^95^ As a result, this chemical transformation functions as a molecular “solubility switch,” enabling enhanced aqueous solubility without altering the peptide’s net charge state.

A representative example is found in the Alzheimer’s-linked amyloid-β (Aβ) sequences, where oxidation of Met35 to Met­(O) disrupts hydrophobic clustering that stabilizes β-sheet formation. This modification significantly reduces aggregation propensity and improves peptide solubility in aqueous environments.^96^ Accordingly, the incorporation or postsynthetic generation of Met­(O) provides a robust approach for converting aggregation-prone hydrophobic segments into soluble, nonionic polar domains.

### 6.3 Selenoxide-Containing Side Chains

Selenomethionine (SeMet), the selenium analogue of methionine, provides a distinctive platform for polarity modulation through oxidation to selenomethionine selenoxide [SeMet­(O)]. Owing to the larger atomic radius and higher polarizability of selenium relative to sulfur, the resulting selenoxide bond (>Se = O) possesses a stronger dipole moment than the corresponding sulfoxide analogue. This enhanced bond polarization increases side-chain hydration and polarity, thereby promoting improved aqueous compatibility within peptide sequences.^97^

In contrast to permanently hydrophilic modifications such as glycosylation, selenoxide formation is intrinsically redox-responsive. SeMet residues can be readily oxidized to the hydrophilic selenoxide state and subsequently reduced back to the hydrophobic selenoether form under mild physiological conditions. This reversible polarity transition enables dynamic regulation of peptide solubility and intermolecular interactions, making selenoxide-containing motifs particularly attractive for the design of stimuli-responsive biomaterials.^98^ In such systems, peptide self-assembly and solubility can be modulated in response to local redox environments, providing a versatile strategy for constructing adaptive peptide-based materials.^97^

### 6.4 Azide-Containing Side Chains

Azide (-N_3_) groups are electrically neutral yet strongly polar, providing handles for bioorthogonal click reactions while remaining fully Fmoc-compatible. Conventional azido-Lys (N_3_K) residues enable SPAAC functionalization but often reduce solubility by removing the ε-ammonium charge. To overcome solubility limitations, a bifunctional azidoamine amino acid was designed to combine an azide handle with a secondary amine within a single side chain.

The synthesis started from Fmoc-allyl-Gly-OH, which was ozonized to form an aldehyde and then underwent reductive amination with 2-azidoethylamine, yielding Nα-Fmoc-Nδ-Boc-protected azidoamine. This monomer was introduced via standard HATU/DIEA or DIC/HOAt activation and remained stable under microwave-assisted Fmoc conditions. Peptides containing the residue showed synthetic yields of 80–91% and purities >90% after RP-HPLC. Hydrophilicity tests revealed a marked retention-time reduction (Δt_R_ = 1.4 vs 7.25 for N_3_K), confirming significantly improved solubility. The side-chain amine remains unprotonated during synthesis but protonates in aqueous media, giving conditional cationic behavior while retaining overall neutral framework. This strategic design achieves a synergy between aqueous solubility and chemical functionality.^87^

### 6.5 PEGylated Side Chains

The conjugation of PEG chains is a proven strategy to enhance the water solubility and stability of therapeutic peptides while masking their intrinsic immunogenicity. In the context of Fmoc-SPPS, PEGylation is typically achieved through site-specific incorporation rather than random conjugation.^99^ Canalle et al. highlighted two primary solid-phase strategies: coupling a PEG-functionalized carboxylic acid to the N-terminus of a resin-bound peptide, or the use of preformed PEGylated amino acid building blocks^66,100^ (Figure 2B). For example, Lu and Felix et al. developed a route where specific amino acid side chains (e.g., Lys) were first coupled to a PEG chain and subsequently incorporated into the peptide sequence during elongation. This “building block” approach ensures precise control over the modification site. However, it is noted that the coupling efficiency on solid support can be limited by steric hindrance; for instance, while unhindered amino acids like Gly are easily functionalized, coupling large PEG chains to sterically hindered residues (e.g., Ile) often requires optimized conditions or lower molecular weight polymers.^100,101^

Furthermore, the molecular weight of the PEG chain requires careful optimization to balance physicochemical properties with biocompatibility. A comparative investigation by Pham Le Khanh et al.^102^ highlights that while low molecular weight PEGs (<600 Da) can induce significant cytotoxicity and hyperosmolality, increasing the chain length excessively may compromise solubility and alter cellular uptake mechanisms. Consequently, a molecular weight range of approximately 800–1000 Da is often suggested as an optimal window, avoiding the toxic effects of short oligomers while maintaining the desirable solubility profile that diminishes with longer polymer chains.

### 6.6 PASylation Side Chains

An emerging bioinspired alternative to PEGylation is PASylation, a strategy utilizing random coil polypeptide sequences comprising uncharged residues Pro, Ala, and Ser (PAS). These sequences are rationally designed to adopt a stable random coil conformation in aqueous solution, thereby significantly expanding the peptide’s hydrodynamic volume to retard renal clearance (Figure 4).^103^ The specific incorporation of Pro prevents secondary structure formation through entropic stiffness, while the hydrophilic amide backbone and Ser hydroxyls ensure high water solubility without introducing net charge.^104^ In the context of Fmoc-SPPS, PASylation offers distinct advantages over traditional synthetic polymers. Unlike chemical PEG reagents which are often polydisperse, PAS sequences are assembled using standard Fmoc-Pro-OH, Fmoc-Ala-OH, and Fmoc-Ser­(tBu)–OH monomers. This allows for the precise synthesis of monodisperse, sequence-defined solubilizing tags with exact control over chain length and hydrophilicity. Furthermore, PAS polypeptides are biodegradable and do not elicit antipolymer antibodies, addressing the critical issues of tissue accumulation and immunogenicity often associated with high-molecular-weight PEG.

**Figure 4:**
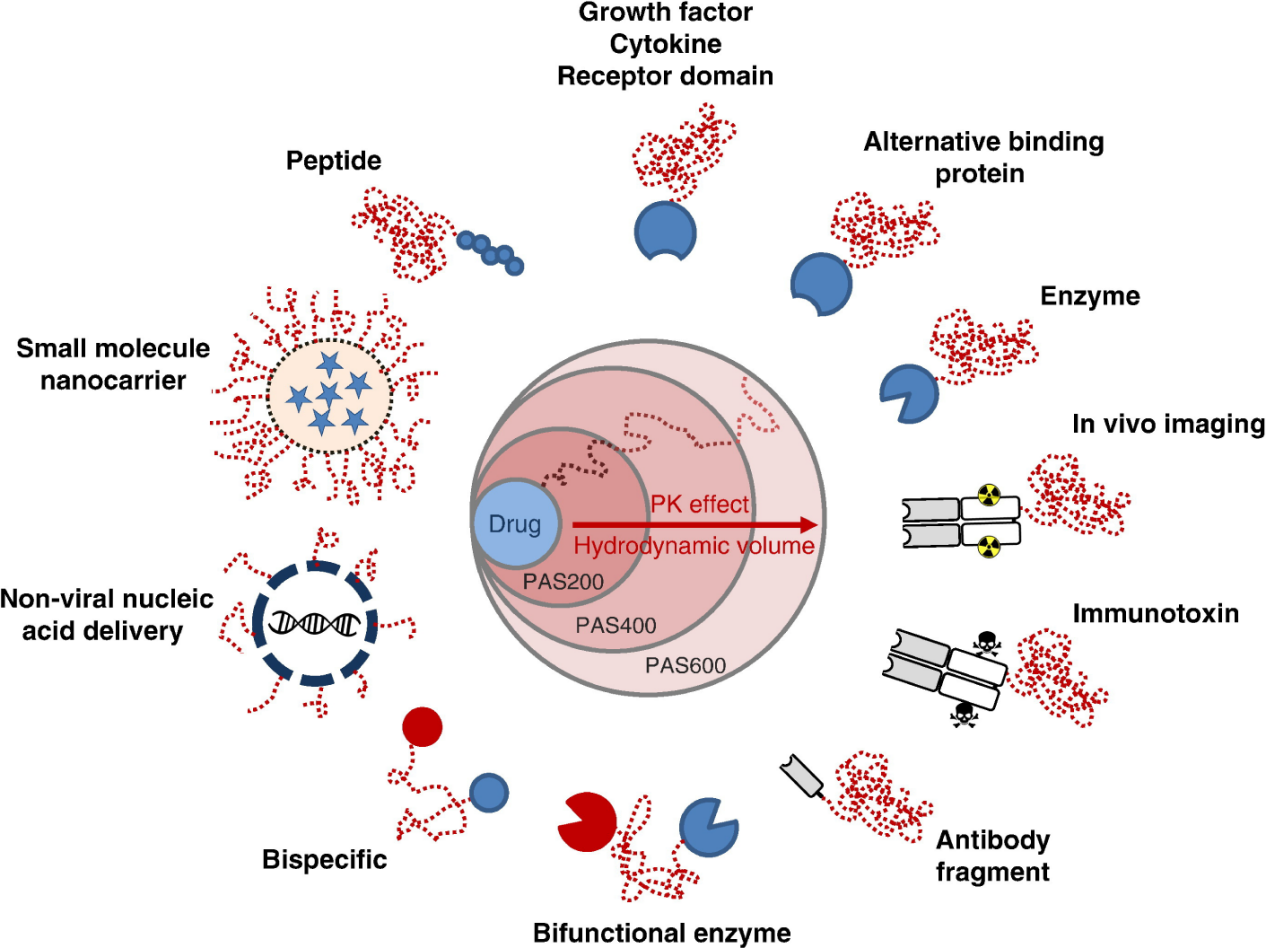
Therapeutic versatility of PASylation. Mechanism: Elongating the PAS sequence increases hydrodynamic volume, thereby retarding renal clearance. Applications: This half-life extension strategy is applicable to a broad spectrum of therapeutics, ranging from peptides, enzymes, and proteins to antibody fragments and nanocarriers for imaging or targeted therapy.^103^ Copyright 2017 Elsevier.

### 6.7 Concluding Remarks

Sugar- and azide-derived polar nonionic amino acids provide complementary strategies for enhancing solubility and synthetic manageability in Fmoc-SPPS. Sugar side chains form multivalent hydroxyl networks that stabilize hydration shells, whereas azidoamine residues combine polarity with orthogonal reactivity. Both classes endure repetitive base and acid treatments and yield monomeric, processable peptides even at high concentrations. Future efforts may explore hybrid polar architectures-such as azide-terminated glycans or PEG-extended sugars- to achieve synergistic improvements in solubility, reactivity, and biocompatibility. These developments will extend the utility of polar nonionic residues in designing multifunctional peptides for biomedical, diagnostic, and material applications.

## 7 Challenges

Although a wide range of Fmoc-compatible ncAAs have been developed to enhance peptide solubility, several practical and conceptual limitations remain in both monomer preparation and solid-phase synthesis. These challenges arise from the intrinsic physicochemical properties of hydrophilic side chains, the constraints of resin-based chemistry, and the analytical difficulties associated with polar or highly charged sequences. Gaining insight into these constraints is critical for the strategic development of advanced solubility-enhancing building blocks.

### 7.1 Poor Compatibility with SPPS Solvents

Many ncAAs designed to enhance aqueous solubility, particularly those carrying bulky protecting groups, glycans, phosphorylcholine moieties, sulfonates, or extended PEG chains, could exhibit limited solubility in the standard (i.e., DMF) or greener SPPS solvent systems, defined in Section 2.^27,36^ Limited dissolution of Fmoc-ncAA monomer lowers their effective concentration during coupling, promotes precipitation during activation, and ultimately reduces coupling efficiency. This is especially problematic for highly substituted sugar derivatives and zwitterionic residues.^63^ To address this limitation, recent studies have demonstrated that the poor solubility of these hydrophilic Fmoc-ncAAs can be effectively alleviated by performing SPPS using binary solvent mixtures, such as DMSO/1,3-dioxolane, DMSO/2-MeTHF, or DMSO/ethyl acetate . These combinations balance polarity and viscosity to enhance Fmoc-ncAA dissolution and resin swelling, critical for coupling efficiency during SPPS synthesis.^105,106^

### 7.2 Inefficient Coupling and Resin-Associated Side Reactions

Paradoxically, the incorporation of these solubilizing residues is often hindered by the very physicochemical properties they are designed to impart. For instance, the substantial steric bulk of glycosylated or PEGylated amino acids can severely impede reagent access to the N-terminus, significantly lowering coupling kinetics and leading to deletion sequences.^100^ Furthermore, the introduction of multiple polar or charged residues can occasionally promote sequence-dependent secondary structure formation and intramolecular interactions, leading to distinct aggregation or resin collapse on the solid support. Specific side-chain reactivities also pose critical challenges beyond the reported overacylation of imidazolium residues and the lactamization risks of Fmoc-Dab derivatives.^64^ Notably, histidine derivatives are highly prone to racemization during activation,^107^ aspartic acid containing sequences are prone to aspartimide formation under basic conditions, and arginine residues can undergo intramolecular cyclization to generate arginyl lactam byproducts.^108^ These side reactions are all exacerbated under the repeated basic conditions employed for Fmoc deprotection.^109^

### 7.3 Epimerization and Chemical Instability of Modified ncAAs

Several solubility-enhancing ncAAs are chemically fragile under basic (piperidine) or acidic (TFA) conditions. Racemization, β-elimination,^24^ phosphate or sulfate hydrolysis,^23,75^ and protecting-group cleavage can occur during SPPS, reducing the effective incorporation of the desired residue. These instabilities are particularly relevant to phosphorylated, sulfated, glycosylated, and backbone-modified monomers^88^ and sulfonium-based side chains, which necessitate postsynthetic alkylation strategies to avoid degradation under basic conditions.^65^

### 7.4 Limited Commercial Availability and High Cost of Polar ncAAs

Many hydrophilic ncAAs-such as PEGylated residues, glycoamino acids, phosphorylcholine-bearing building blocks, or taurine-based analogues, are expensive, require multistep synthesis, or are commercially unavailable. Their preparation often involves orthogonal protecting group strategies, phosphoramidite or glycosylation chemistry, or triphosgene activation, which increases cost and limits accessibility for routine peptide synthesis.^64,65^

### 7.5 Structural Heterogeneity Introduced by Large Hydrophilic Modifications

While PEGylation, glycosylation, and zwitterionic modifications improve solubility, they may generate microheterogeneity in the final peptide due to the polydisperse nature of polymer chains.^32,100^ Variability in PEG chain length, partial glycan loss, or incomplete side-chain installation can alter biological activity, disrupt secondary structure, and complicate analytical evaluation. These issues reduce reproducibility and require more sophisticated characterization tools.

### 7.6 Purification and Analytical Bottlenecks for Highly Polar Peptides

Peptides bearing multiple hydrophilic or charged residues often behave atypically on reverse-phase HPLC, displaying minimal retention,^87^ extensive peak broadening, or coelution with impurities. Extremely polar products may elute near the void volume, making purification challenging and reducing isolated yields.^64^ A prominent analytical challenge lies in the separation of full-length peptides from their closely related impurities, particularly deletion sequences (e.g., n-1 species). D’Hondt et al. highlighted that when a missing amino acid contributes minimally to the overall hydrophobicity, common in highly charged or polar sequences, the resulting impurity often coelutes with the main product on reverse-phase HPLC.^110^ This critical limitation underscores the need for complementary analytical techniques, such as ion-exchange chromatography or hydrophilic interaction liquid chromatography, for the accurate purity assessment of highly hydrophilic peptides.^111,112^ These orthogonal methods effectively resolve deletion sequences that otherwise coelute on C_18_ columns, ensuring higher product integrity.

### 7.7 Immunogenicity and ADME Concerns Associated with Non-Natural Modifications in Clinical Translation

Although hydrophilic modifications improve solubility, they may introduce immunogenic epitopes or alter pharmacokinetic profiles. PEGylation can trigger anti-PEG antibodies;^35^ non-native glycoforms may affect immune recognition; and highly charged or zwitterionic motifs can modify renal clearance or biodistribution. Indeed, we should note that molecular weight itself influences immunogenic risk: small peptides with molecular weights in the 1–2 kDa range are typically not strongly immunogenic, whereas molecules exceeding roughly 10 kDa begin to elicit more robust immune recognition.^113,114^ These considerations constrain the design of solubilizing groups for therapeutic peptides.

In summary, while solubility-enhancing Fmoc-ncAAs substantially expand the design space for chemically synthesized peptides, challenges in monomer solubility, synthetic stability, coupling efficiency, and analytical characterization remain significant barriers. Addressing these limitations will require progress in protecting-group chemistry, resin engineering, solvent optimization, and scalable monomer synthesis. Continued innovation in these areas will be essential for fully realizing the potential of ncAA-based side-chain engineering in modern peptide science.

## 8 Outlook

To bridge the gap between current limitations and the full potential of Fmoc-ncAAs, future research must converge on chemical, material, and digital frontiers. The following strategies offer tangible solutions to the challenges outlined above:

### 8.1 Solvent and Resin Engineering

Overcoming monomer solubility issues and coupling inefficiencies necessitates the broader adoption of “green” high-swelling solvent systems and superior resin matrices. While traditional DMF/NMP systems are effective, binary mixtures such as DMSO, GVL, or 2-MeTHF combined with polar cosolvents—have shown capability in disrupting interchain hydrogen bonds that drive aggregation.^53,115,116^ Furthermore, the transition from polystyrene-based supports to totally PEGylated resins (e.g., ChemMatrix) is critical. However, as ChemMatrix is no longer commercially viable,^117^ the field has shifted toward alternative green supports.^118^ Notably, polyethylene glycol-polyacrylamide resins have been revitalized through a sustainable synthesis in recyclable silicone oil, replacing hazardous CCl_4_. These modern beads are spectroscopically transparent and exhibit “stealth” characteristics, offering superior solvation properties and higher swelling volumes in both organic and aqueous media, thereby minimizing steric hindrance and allowing reagents to penetrate complex, aggregated sequences more effectively.^118^

### 8.2 Advanced Synthetic Strategies

To mitigate the chemical instability of sensitive ncAAs, research should focus on chemoenzymatic ligation strategies or the design of highly labile orthogonal protecting groups.^64^ Addressing this urgent need, novel photocatalytic platforms based on pyridinemethyl and picoc chemistries have recently emerged. These strategies utilize visible light to trigger C-heteroatom bond cleavage, enabling an acid-free and TFA-free deprotection process that not only suppresses aspartimide formation but is also compatible with aqueous and green solvent workflows.^119,120^ Furthermore, two-step or postassembly conjugation strategies are becoming increasingly attractive. These modular approaches, such as click chemistry, oxime ligation, or enzymatic glycosylation, allow the introduction of polar or bioorthogonal side chains after peptide assembly, bypassing steric barriers during Fmoc-SPPS. In addition, on-resin incorporation of multiple distinct bioorthogonal functionalities enables conditional orthogonal reactions, facilitating multicyclization and the construction of structurally complex peptide architectures.^121^ Finally, microwave-assisted SPPS strategy can further enhance coupling efficiency during assembly.^46^

### 8.3 Precision Material Design

Addressing the heterogeneity and immunogenicity of solubilizing tags lies in the shift toward discrete, monodisperse oligomers and novel zwitterionic motifs. Unlike traditional polydisperse PEGs, discrete PEGs (dPEGs) with defined chain lengths eliminate molecular weight variability, ensuring reproducible pharmacokinetic profiles and simplifying regulatory characterization. Concurrently, the exploration of nonimmunogenic zwitterionic polymers, such as poly­(sarcosine), phosphorylcholine, or carboxybetaine analogues, offers a superior alternative.^35,100^ These materials generate a superhydrophilic hydration shell that not only enhances solubility but also resists nonspecific protein adsorption (fouling), potentially reducing the risk of antidrug antibody formation observed with repeated PEG administration.

### 8.4 Digital and Analytical Innovation

To resolve analytical bottlenecks, the integration of advanced chromatography and computational intelligence is essential. Techniques such as Ultra-High Performance Liquid Chromatography coupled with Ion Mobility Mass Spectrometry provide the resolution needed to separate complex deletion sequences and conformers that coelute in standard HPLC.^110^ Furthermore, the application of AI-driven computational modeling, utilizing molecular dynamics simulations and machine learning algorithms (e.g., Aggrescan or AlphaFold-based tools), will revolutionize sequence design. These tools can predict aggregation-prone regions *in silico*, enabling the rational, presynthetic selection of the optimal ncAA type and placement. This shift moves the field from empirical trial-and-error to precision engineering, ensuring solubility is designed into the molecule from the start.^122^

## 9 Conclusion

The rational design of Fmoc-based ncAAs has emerged as a versatile strategy to overcome limited solubility, one of the long-standing challenges in peptide chemistry. By systematically modifying side-chain functionalities, researchers have developed charged, polar, and zwitterionic analogues that significantly improve synthetic-phase manageability and solution-state behavior. Collectively, these design principles have advanced the field from empirical optimization toward molecular-level control of peptide solubility, stability, and processability.

Despite this progress, several challenges remain. The stability of hydrophilic side chains, particularly glycosylated, PEGylated, or zwitterionic motifs, under strongly acidic cleavage and deprotection conditions remains a bottleneck for long-sequence synthesis. Similarly, steric hindrance and coupling inefficiency introduced by bulky or extended substituents often lead to incomplete sequences or resin aggregation. Addressing these issues will require the development of acid-stable protecting groups, orthogonal cleavage strategies, and advanced resin architectures that maintain reactivity while minimizing side reactions.

To further expand the structural diversity and functional utility of ncAAs, two-step or postassembly conjugation strategies are becoming increasingly attractive. These modular approaches, such as click chemistry, oxime ligation, or enzymatic glycosylation, allow the introduction of polar or bioorthogonal side chains after peptide assembly, bypassing steric barriers during Fmoc-SPPS. Meanwhile, integration into proteins and macromolecular systems represents the next frontier. Semisynthetic methods, such as protein ligation and genetic code expansion, will enable the incorporation of solubility-enhancing residues into larger protein scaffolds, thereby bridging peptide chemistry with protein engineering and synthetic biology.

Looking forward, the convergence of Fmoc-SPPS, postsynthetic modification, and biological conjugation will transform solubility tuning from a synthetic challenge into a rational design element. Combining robust chemical synthesis with enzymatic and bioorthogonal methodologies will enable precise control of hydrophilicity across peptides, proteins, and hybrid biomaterials. Ultimately, these advances will empower the creation of next-generation soluble peptide systems for therapeutics, diagnostics, and functional materials, firmly establishing ncAA-based side-chain engineering as a cornerstone of modern peptide science.
